# Humoral immune response and changes in peritoneal cell populations in rats immunized against two *Leptospira* serovars; serovar patoc and serovar pyrogenes

**DOI:** 10.1186/s12865-023-00574-z

**Published:** 2023-10-17

**Authors:** Dakshika Gangani, Wathsala Dissanayake, Rajiva de Silva, Kaushalya Anuradha, Lilani Karunanayake, Narmada Fernando, Senaka Rajapakse, Sunil Premawansa, Shiroma Handunnetti

**Affiliations:** 1 Institute of Biochemistry Molecular Biology and Biotechnology, University of Colombo, Colombo 03, Colombo, Sri Lanka; 2 Department of Immunology, Medical Research Institute, Colombo 08, Colombo, Sri Lanka; 3 Department of Bacteriology, Medical Research Institute, Colombo 08, Colombo, Sri Lanka; 4 Postgraduate Institute of Medicine, University of Colombo, Colombo 08, Colombo, Sri Lanka; 5 Department of Zoology and Environment Sciences, Faculty of Science, University of Colombo, Colombo 03, Colombo, Sri Lanka

**Keywords:** *Leptospira*, IgM and IgG response, Peritoneal cells, B1 lymphocytes, B2 lymphocytes, Serovar Patoc, Serovar Pyrogenes

## Abstract

**Background:**

Leptospirosis is a zoonotic disease caused by *Leptospira* species. Variations in lipopolysaccharide (LPS) structure in *Leptospira* are known to be associated with the serovar diversity and antigenicity. Development of immunodiagnostics for early detection of leptospirosis based on immune responses against different pathogenic antigens as well as development of vaccines are important. Hence, this study has assessed the immune response generated against leptospiral LPS and whole antigen preparations of pathogenic and saprophytic *Leptospira* and specific changes in peritoneal cells was also studied to elucidate the cellular responses associated with immune response of Wistar rats.

**Methods:**

During the study, immune response induced by two types of *Leptospira* antigen preparations of two selected serovars was compared. Changes in the specific peritoneal cell subpopulations following immunizations of rats were analyzed using flow cytometry.

**Results:**

Of the two antigen preparations tested, the LPS extract induced a higher IgM immune response as opposed to the sonicated antigen preparation. Of the two serovars tested, *L. interrogans* serovar Pyrogenes had induced a higher IgM response compared to that by *L. biflexa* serovar Patoc. Considering the IgG titers, equivalent responses were observed with all four antigen preparations. Significant increases in lymphocytes were observed following immunization with LPS of both serovars. Interestingly, the B2 cell percentages increased significantly during the immunization period. Further, significant correlations were observed with both IgM and IgG responses and percentage of B2 cells in the peritoneal cavity (PC).

**Conclusion:**

LPS extract of *L. interrogans* serovar Pyrogenes induced higher IgM response while the IgG response was equivalent among the four antigen preparations tested. Significant increase of B2 cell percentage in the peritoneal cavity during the immunization reflects the accumulation of B2 cells in the PC which may play considerable role in generating humoral response against *Leptospira* antigens.

**Supplementary Information:**

The online version contains supplementary material available at 10.1186/s12865-023-00574-z.

## Introduction

Leptospirosis is a zoonosis which is caused by *Leptospira.* It is worldwide distributed, being more common in the tropical and subtropical regions where factors for its transmission are particularly favorable. Estimated annual global incidence of leptospirosis is 1.03 million cases (95% CI 434,000–1,750,000) that results in approximately 2.90 million Disability Adjusted Life Years lost per annum as well as 58,900 deaths (95% CI 23,800–95,900) [1, 2]. Wide distribution of *Leptospira* species is due to many reasons including the ability of the pathogen to colonize the renal tubules of a diverse range of animals, ability to survive in different environmental conditions, sometimes even as free-living organism with differential expression of protein which involve in adapting to different conditions [3]. The underlying mechanisms of host immunity to *Leptospira* as well as factors contributing to the leptospirosis pathogenesis are still being analyzed [4]. In Sri Lanka, the estimated annual incidence of leptospirosis is 300 (95% CI 96.54–604.23) per 100,000 population [5]. Males are predominantly affected comprising approximately 80% of the total burden of disease both globally as well as in Sri Lanka [1, 6].

Leptospires were classified according to their antigenic determinants. Molecular classification has divided the *Leptospira* genus into several species based on the DNA relatedness, while the serological classification based on antigenic relatedness between isolates defines the serovars [7]. Antigenic diversity among the *Leptospira* serovars mainly occur due to the variations in the carbohydrate side chains of lipopolysaccharide (LPS) [3]. The expression of the surface-exposed epitopes in a mosaic of the lipopolysaccharide antigens determine the serovar classification and specificity of these epitopes are due to their sugar composition and orientation [4, 8]. *Leptospira* genus was traditionally divided into two groups as saprophytes - *Leptospira biflexa sensu lato -* and pathogens - *Leptospira interrogans sensu lato -* based on their virulence [9]. With recent phylogenetic analysis *Leptospira* have been divided in to three lineages according to the level of pathogenicity of the species as saprophytic, intermediate, and pathogenic [10, 11].

Identification of leptospiral proteins expressed during infection might be the targets for host immune response and may be important in immunodiagnosis as well and tracing the biomarkers of pathogenesis [2]. Antibodies that recognize several protein antigens from the outer membrane, periplasmic space, and the inner membrane as well as serovar specific lipopolysaccharide have been detected in serum samples taken from Leptospirosis infected patients [12]. Such immunogenic proteins which elicit natural immune responses as well as following immunization of experimental hosts with leptospiral antigens can be considered as targets of immunodiagnosis. The identification of protein antigens which are cross-species conserved or cross serovar conserved are important in this context [1]. However, there is a limited knowledge available on the variability of humoral response with respect to the infecting serovar strain [13]. Regardless of the way of infection, it is very important to have an analysis of antibody response especially in diagnostics and as well as in determination of severity. It will provide insights into mechanisms involved in pathogenesis and will further provoke the knowledge for the development of therapeutics [8, 13, 14].

The selection of a route of injection for experimental immunization to induce an immune response or experimental inoculations of infections is made based on several considerations [15, 16]. A recent study has shown a highly effective defense against viral infection due to immunological responses induced in the mouse peritoneal cavity [16]. Another recent study in rainbow trouts (*Oncorhynchus mykiss*) has also shown to induce effective humoral and cellular immune responses following intraperitoneal vaccination [17]. Peritoneal cavity harbors different types of immune cells including macrophages, B cells and T cells. B1 cells generated from fetal liver hematopoietic stem cells (HSCs), are positively selected by self-antigens and home predominantly to peritoneal cavity (PC). B1 cells play a major role in producing IgM creating first line of protection against number of pathogens. Further, B1 cells express receptors which are shown poly specific and often have a preference to common bacterial polysaccharides. Of the two subtypes, B1b cells which is more commonly involved in antibody response appear to recognize more types of antigens without T helper cells [18–20]. CD5 + B1a cells produce IL-10 and due to this secretion, these cells possess different kinds of regulatory properties involving innate immunity, autoimmunity and immune regulation. Subpopulations of mouse and human B-1a cells express CD5 receptors and all rabbit peritoneal cells are CD5 positive whereas CD5 is not detectable in rats [21, 22]. Studies using both naïve and antigen exposed B cells on B cell migratory patterns have shown that transit through peritoneal cavity modifies their migratory capacity, the splenic B2 cells adoptively transferred into the peritoneal cavity gain the ability to reenter this site and these patterns are dependent on expression of a variety of chemokines [23].

In the present study, we have investigated the humoral immune response against sonicated *Leptospira* antigen preparations and LPS purified from two *Leptospira* serovars; serovar Patoc and serovar Pyrogenes. Further, identification of specific peritoneal cell sub populations were performed in parallel to determine the changes that occurred in immune cells harvested from the peritoneal cavity in order to correlate the changes in B cell populations with the IgG and IgM response.

## Results

### IgM response against*L. biflexa* serovar Patoc and *L. interrogans* serovar Pyrogenes

**Fig. 1:**
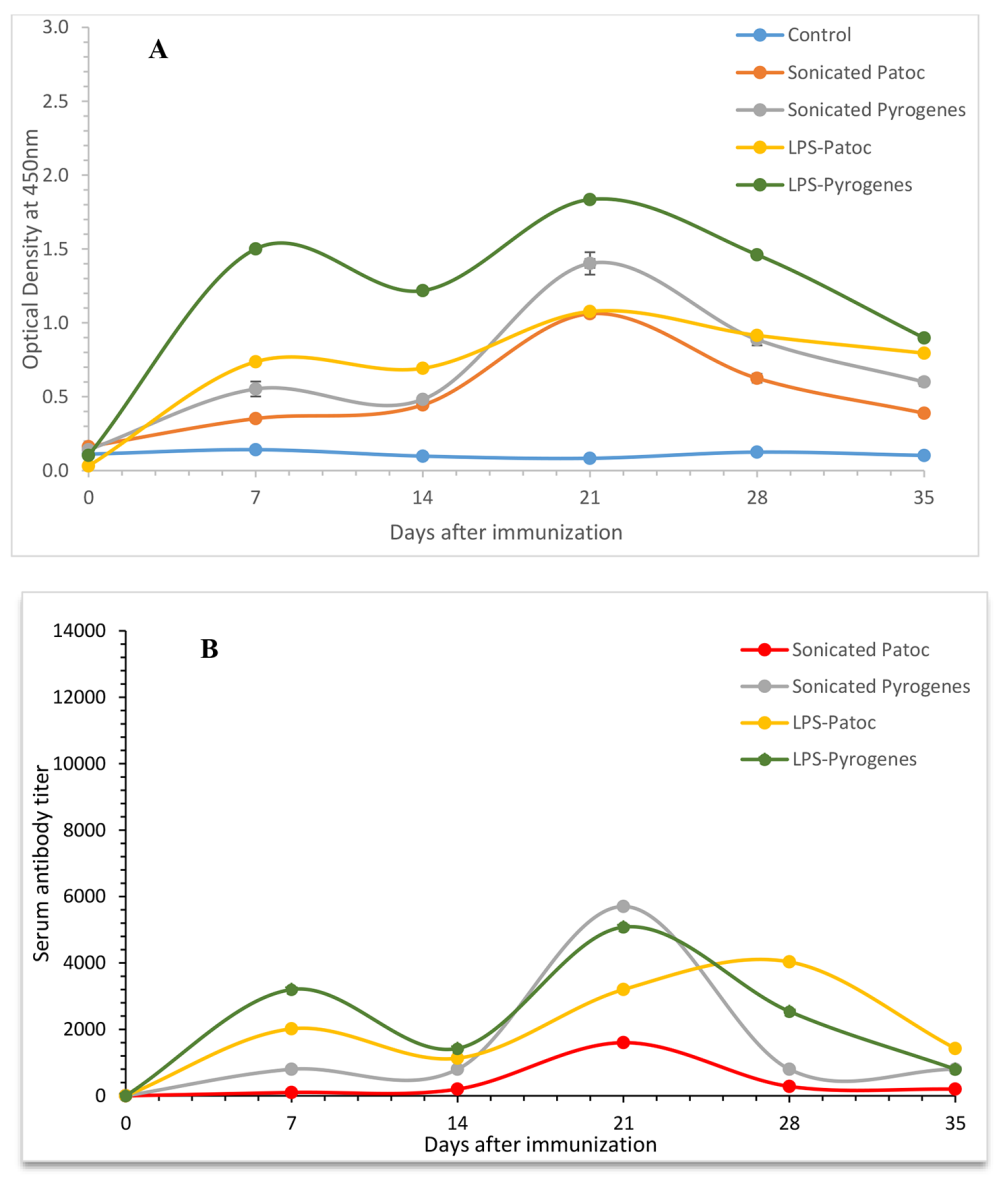
IgM response as measured as OD 450 nm of 1:100 serum dilution **(A)** and IgM titers **(B)** of rats immunized with sonicated preparations of *Leptospira biflexa* serovar Patoc, and *L. interrogans* serovar Pyrogenes and LPS extracted from *L. biflexa* serovar Patoc and *L. interrogans* serovar Pyrogenes (Each data point represents the mean ± SEM, n = 6)

As depicted in Fig. 1A, IgM response increased and fluctuated compared to day 0 with two peaks on day 07 and day 21, having the highest IgM level on day 21 in all four groups (p *<* 0.001*).* Similarly, the IgM titers also showed two peaks on the same days except for LPS-Patoc which showed peak at day 28 (Fig. 1B). Of the four-antigen preparation compared, even though LPS-Pyrogenes showed higher OD 450 nm values from day 7 to 28, the titers in all four groups were comparable throughout the period tested.

The initial IgM response induced by LPS of both serovars by day 7 was significantly higher than that induced by the sonicated antigen (p *<* 0.001*).* IgM response against LPS continued to be significantly high (p *<* 0.001*)* except on day 21 in serovar Patoc. OD 450 nm at 1:100 serum dilutions indicated that both LPS (p *<* 0.001) and sonicated (p *<* 0.001) preparations of serovar Pyrogens had induced higher IgM response compared to those by the serovar Patoc preparations throughout the period tested except on day14.

### Anti - *Leptospira* IgG response and IgG serum antibody titers

**Fig. 2:**
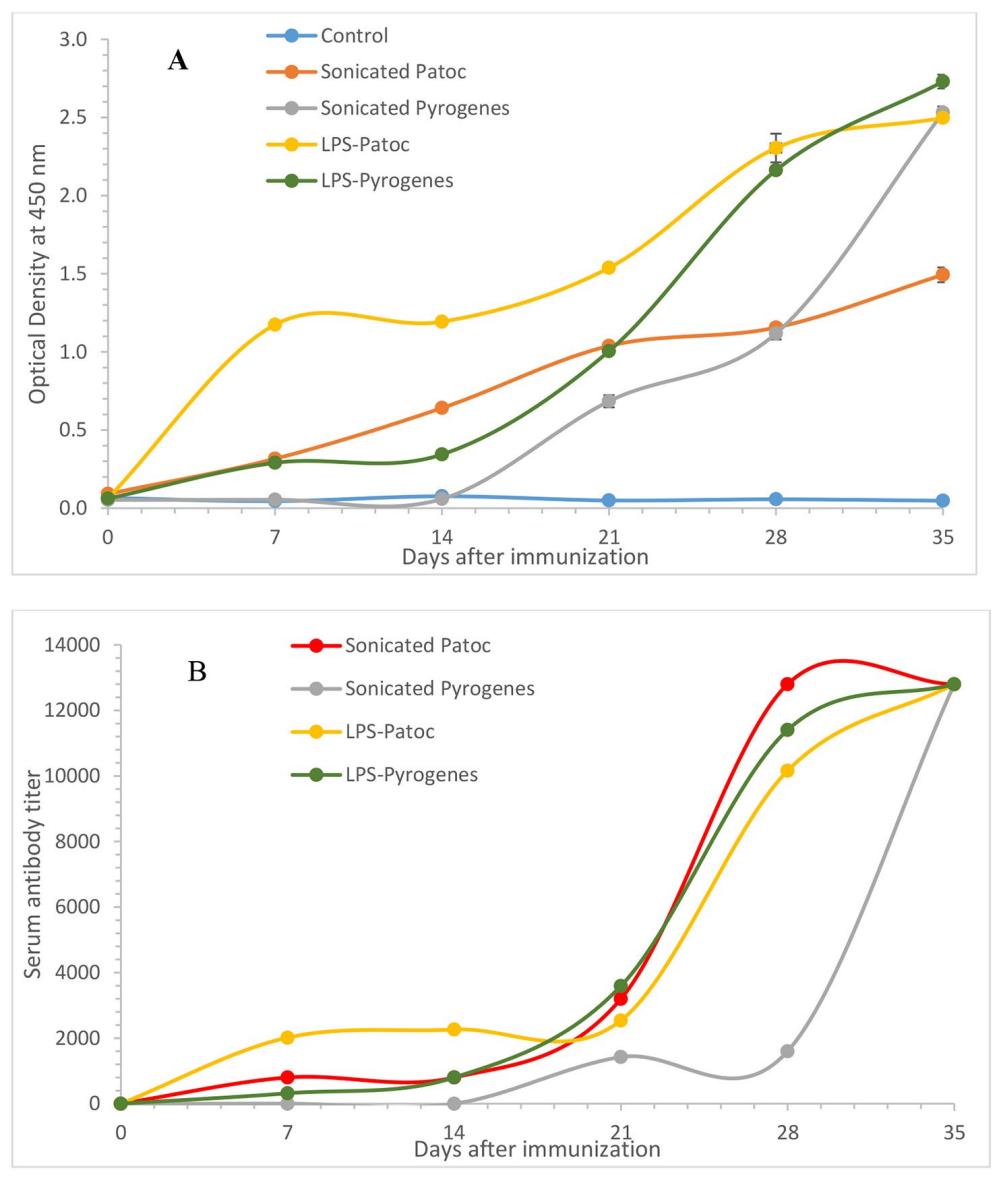
IgG response as measured as OD 450 nm of 1:100 serum dilution **(A)** and IgG titers **(B)** of rats immunized with sonicated preparations of *Leptospira biflexa* serovar Patoc, and *L. interrogans* serovar Pyrogenes and LPS extracted from *L. biflexa* serovar Patoc and *L. interrogans* serovar Pyrogenes (Each data point represents the mean ± SEM, n = 6)

IgG response as measured by OD 450 nm in 1:100 serum dilution (Fig. 2A) and IgG titers (Fig. 2B) increased continuously up to day 35 with respect to all four antigen preparations: sonicated serovar Patoc, sonicated serovar Pyrogenes, LPS-Patoc and LPS-Pyrogenes. Immune response induced by both preparations of Patoc and LPS- Pyrogenes significantly increased from day 7 onwards except sonicated pyrogenes which showed a delayed response. However, Pyrogenes sonicated preparation reached the same titer as the other preparations by day 35 (p *<* 0.001).

Even though at early-stage rats immunized with both preparations of serovar Patoc indicated comparatively high IgG levels as detected by OD 450 nm at 1:100, subsequently IgG level of rats immunized with serovar Pyrogenes exceeded that of the Patoc by day 35 (p *<* 0.001). This trend was not detectable with IgG titers.

### Sub population changes in peritoneal cells following immunization with *Leptospira* LPS

**Fig. 3:**
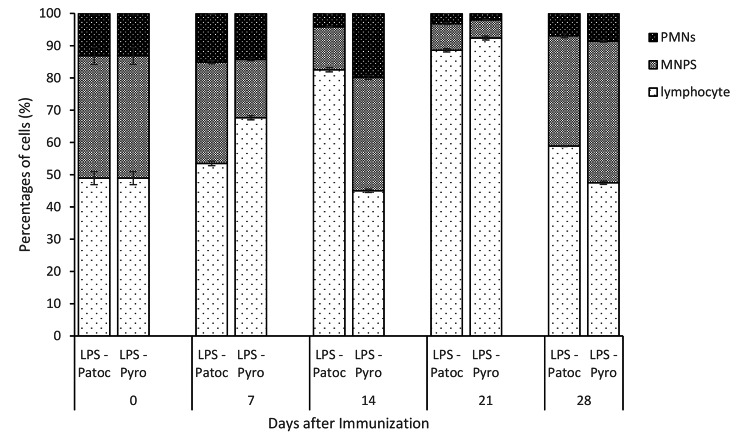
Changes in different types of peritoneal cells, lymphocytes, mononuclear phagocytes (MNPs) and polypmorphonuclear cells (PMNs) of rats immunized with the two LPS preparations, *Leptospira biflexa* serovar Patoc (LPS-Patoc) and *Leptospira interrogans* serovar Pyrogenes (LPS-Pyro).

Variation in immune cell subpopulations present in the peritoneal cavity of rats immunized with LPS-Patoc and LPS-Pyrogenes were analyzed with respect to the percentages of mononuclear phagocytes (MNPs), granulocytes, lymphocytes, T cells and B1 and B2 cells. Prior to immunization, lymphocytes represented 48.9% and it increased to significantly higher levels in Patoc immunized rats (82.6% and 88.6% by day 14 and 21 respectively; p < 0.001) whereas in Pyrogenes immunized rats reached 67.7% and 92.4% by day 7 and 21 respectively; p < 0.001 (Fig. 3).

**Fig. 4:**
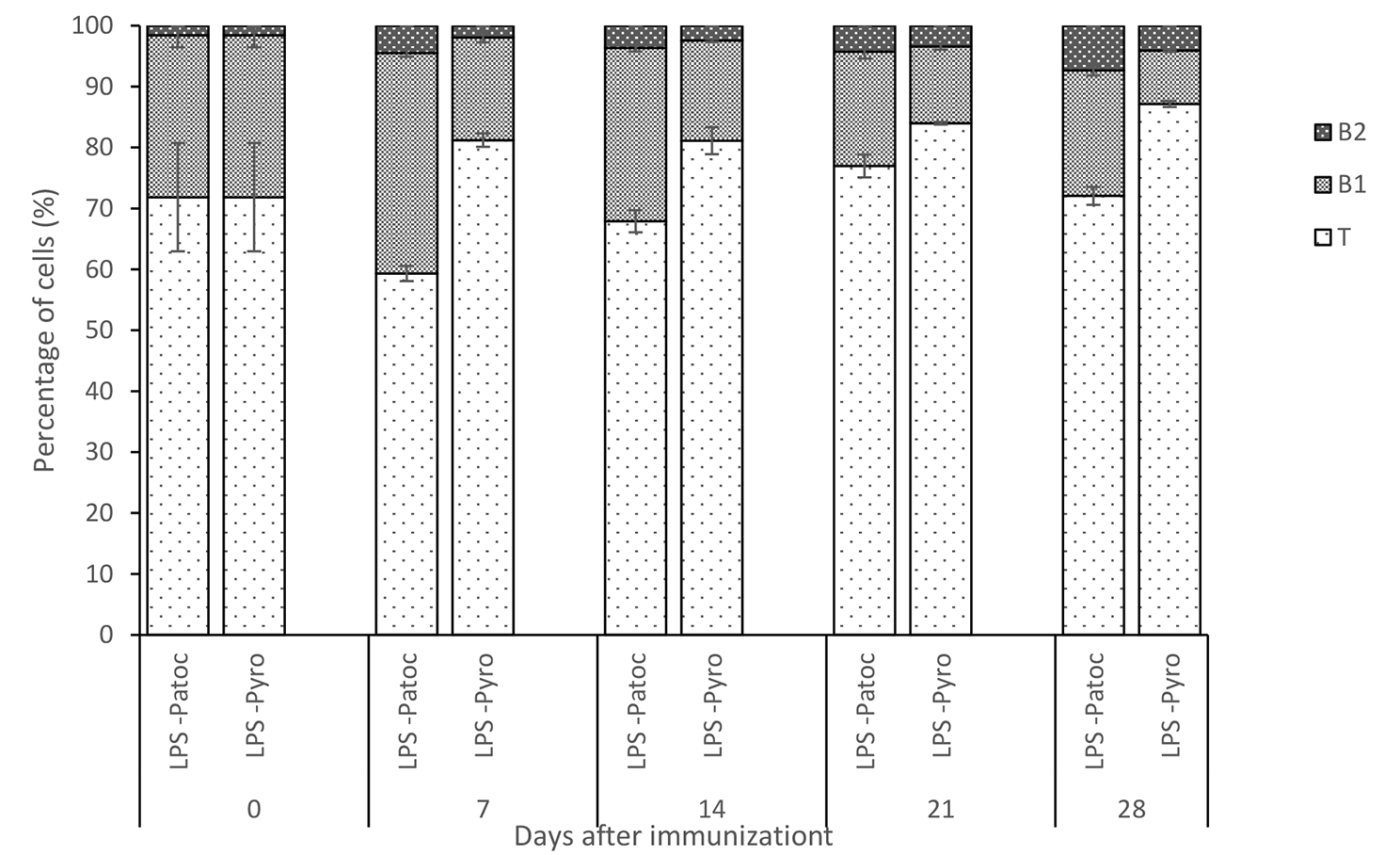
Changes in different peritoneal lymphocyte subpopulations, T, B1 and B2 cell of rats immunized with the two LPS preparations, *Leptospira biflexa* serovar Patoc (LPS-Patoc) and *Leptospira interrogans* serovar Pyrogenes (LPS-Pyro).

As indicated in Fig. 4, the T cell percentage was significantly high in LPS-Pyrogenes immunized rats over LPS-Patoc immunized rats throughout the period despite the fluctuation of lymphocyte counts (p < 0.001) (Supplementary Table 1). Within the lymphocyte population in the peritoneal cavity, changes in T cells (CD3^high^) were not significant although there was a trend in its increase (Fig. 4). With respect to B1 cell subpopulation (CD45RA+; CD11b^mid^), in rats immunized with LPS- Patoc the B1 cells increased from 26.6 to 36.2% by day 7 (p < 0.001) and decreased thereafter. However, in rats immunized with LPS-Pyrogenes, the B1 cells decreased continuously from 26.6% up to day 28 to reach a very low level (8.8%; p < 0.001) and their percentages were significantly lower compared to that of LPS-Patoc immunized rats in throughout the immunization period (p < 0.001). In contrast in both LPS-Patoc and LPS-Pyrogenes immunized rats, B2 cells (CD45RA+; CD11b^low^) increased significantly from 1.6 to 7.3% and 4.1% respectively (p < 0.001) showing an inverse relationship compared that of B1 cells.

### Correlation between IgM and IgG response and peritoneal B cell subpopulations

We compared the IgM and IgG response (OD 450 nm of 1:100 serum dilution and the antibody titers) in LPS-Patoc and LPS-Pyrogenes immunized groups to see whether there is relationship between the immune response against *Leptospira* LPS and the changes observed in B cell subpopulations in the peritoneal cavity. Interestingly, the B2 subpopulations showed significant correlation with both IgM and IgG response as measured by both the OD 450 nm of 1:100 serum dilution and antibody titers of the two groups of rats immunized with LPS extracts of the two serovars (Table 1). Further, no such associations were observed of the percentages of T cells with IgM or IgG response as measured by the two criteria mentioned above.

**Table 1:** Association between IgM and IgG response and peritoneal lymphocyte subpopulations in rats immunized with sonicated antigens and LPS of the two serovars tested

Comparison	OD 450 nm of 1:100 serum dilution		Antibody titer	
	Significance*		Significance†	
B1 cells with	R	*P*	r	*P*
LPS-Patoc IgM	-0.346	0.061	-0.202	0.284
LPS-Patoc IgG	-0.398	**0.029**	-0.312	0.093
LPS-Pyrogenes IgM	-0.822	**< 0.001**	-0.159	0.400
LPS-Pyrogenes IgG	-0.783	**< 0.001**	-0.918	**< 0.001**
**B2 cells with**				
LPS-Patoc IgM	0.743	**< 0.001**	0.644	**< 0.001**
LPS-Patoc IgG	0.953	**< 0.001**	0.823	**< 0.001**
LPS-Pyrogenes IgM	0.641	**< 0.001**	0.513	**0.004**
LPS-Pyrogenes IgG	0.918	**< 0.001**	0.905	**< 0.001**

n = 6; * Pierson correlation, **†** Spearman correlation

## Discussion

In the present study we investigated the IgM and IgG responses, variation in peritoneal cell subpopulation and their associations in response to immunization with two types of preparations of *Leptospira* antigens. Our findings in this study indicated that IgM response appears in the early stage of infection with greater significance subsequently followed by a higher IgG response and more importantly, of the two antigen preparations tested, the *Leptospira* LPS extracts induced a higher IgM immune response as opposed to the sonicated *Leptospira* antigen preparation. Of the two *Leptospira* serovars tested, *L. interrogans* serovar Pyrogenes had induced a higher IgM response compared to that by *L. biflexa* serovar Patoc. Interestingly the IgG titers raised against immunization with four antigen preparations were similar. Considering the different types of lymphocytes present at the site of immunization, there was a significant increase in B2 lymphocytes and this was significantly correlated with both the IgM and IgG response during immunization period.

Findings of the present study is consistent with those from previous study which had shown that polysaccharide antigens such as LPS of pathogenic *Leptospira* induces a high IgM response and the humoral response is mainly directed to the O antigen portion of the LPS and dependent on the length of LPS [24]. The fact that serovar Patoc has a lower immunogenicity has been attributed to LPS of *L. biflexa* serovar Patoc having a much shorter LPS compared to the pathogenic species and serovars [25]. However, recent studies have shown that the length of LPS has no direct influence on humoral response [13, 25]. It has been shown that LPS of serovar Icterohaemorrhagiae, Copenhageni, and Manilae which had equal length had different immune responses indicating that there are other qualitative factors that contribute to immunogenicity. For example, the nature of the LPS structure, whether it is truncated or not is also associated with anti-LPS immune response [13, 25]. Further with respect to IgM response to the two serovars tested, our findings indicated significantly high immune response in Pyrogenes compared to Patoc. This may be due to high immunogenicity of ‘pathogenic LPS’ compared to ‘non-pathogenic LPS’. Therefore, it is more likely that the changes in the structure or composition of the LPS may be the reason for this antigenic diversity, immunogenicity, mechanism of action and further pathogenicity to Leptospirosis.

Previous studies have reported that *Leptospira* LPS mask the protein associated immune response [13]. The sonicated antigen preparation contains a mixture of both *Leptospira* LPS and protein and antigens. This may explain why the IgM response to sonicated antigen was lower compared to the LPS in both serovar Patoc as well as serovar Pyrogenes. The IgM response had shown a peak antibody level by day 21 or day 28, following second immunization and had reached much lower levels by day 35. This indicates that the antigen persistence and antibody production had decreased two weeks after the second immunization indicating the need for multiple immunizations if a higher antibody response required to be maintained in an experimental immunization. A contrasting, sustained IgM response has been associated with some *Leptospira* strains, especially the virulent strains were associated with renal colonization of bacteria in rodent kidney following the *Leptospira* challenge [13]. In this study, heat-killed *Leptospira* antigen was used for immunization and it was hypothesized that the leptospires constantly re-infect the host via urinary shedding or could re-enter the circulation from the proximal tubule and hence give rise to a persistent IgM response. Although the IgM response was decreased by day 35, the IgG response had continued to increase up to day 35 to reach a much higher level. This is important since it indicates that with two immunizations the isotype switch from IgM to IgG had occurred and continued to produce higher level of IgG antibodies. With respect to IgG subclasses, Vernel-Pauillac et al. had reported that IgG1 and IgG3 are elicited against proteins and polysaccharide antigens such as LPS induced class switching to IgG2 [13]. Present study had not assessed the subclasses of IgG which is a limitation in this study.

In the present study, we selected the groups of rats immunized intraperitoneally with *Leptospira* LPS to determine the immune cells that were present at the site of immunization. On day 0, prior to immunization, lymphocyte population consisted mainly CD3^high^ T cells (71.8%). Of the B lymphocytes, B1 cells were the dominant (26.6%; CD45RA^+^ CD11b^mild^) and a very low level of B2 cells (1.6%; CD45RA^+^ CD11b^low^). At the pre-immune stage the B1 cells were 15-fold higher compared to B2 cells. This observation is consistent with those from previous studies performed in other healthy murine species, BALB/c mice which had reported that the main B cell population in the peritoneal cavity is primarily B1 cells which are self-replenishing [16, 19]. Interestingly, in both LPS-Patoc and LPS-Pyrogenes immunized rats there was a significant increase in B2 cells 1.6 to 7.3% and 4.1% respectively (p < 0.001) showing an inverse relationship compared that of B1 cells. This observation is highly pertinent in consideration of the B cell population that contribute to the antibody production and also for the isotype switch in the immune response. Therefore, the fact that the relative contribution of B1 and B2 B cells to antibody production not being determined is a limitation of the present study. Our previous study on the immunization of rats with a protein antigen (BSA) along with an immunomodulatory polyherbal formulation provides indirect but corroborative evidence on the relative contribution of peritoneal B cells in antibody production and specific expression of mRNA for IgM, IgG and IgA as well the peritoneal B and T cells in expression of costimulatory molecules that lead to T-dependent B cell activation (30 and unpublished data). These data are supportive on the requirements and the process that may occur leading to isotype switching from IgM to IgG. Therefore, it is important to carry out future studies on the immunoglobulin expression studies using peritoneal B cells following *Leptspira* LPS immunization. The gradual, but significant increase in B2 cells present in the peritoneal cavity of the groups of rats immunized with serovar Patoc and serovar Pyrogenes raise the question as to whether it was due to specific recruitment/migration of B2 cells from adjoining tissues to peritoneal cavity or conversion of B1 cells in the peritoneal cavity to B2 cells. In contrast with B1 cells, B2 cells are known to rapidly circulate throughout the peritoneal cavity, the spleen, and other lymphoid compartments [23] and this feature of B2 cells may have contributed to the increase in B2 cells observed during the immunization period in the present study. Interestingly, a recent study conducted using a C57BL/6J mouse model has demonstrated that the G protein-coupled receptor 183 (GPR183) is a chemotactic receptor that regulates the migration of B cells [26]. Further, it has shown that GPR183 is not essential for the accumulation and functions of B1 cells in the peritoneal cavity but it has positive influence on the abundance of B2 cells. It is postulated that this receptor plays a sub-dominant role to that of other chemotactic receptors, such as CXCR5 and CCR7, similarly to the role it plays in regulating immune cell migration. In another study in which inflammation was induced in the peritoneal cavity of BALB/c mice using Pristane oil injection migration of subpopulations B1 and B2 cells has been proposed to explain the decrease in B cells [27]. This study has further elaborated that the B1 cells migrated from the peritoneal cavity to the mesenteric lymph nodes where they differentiate into antibody secreting plasma cells. Immune cells also migrate to the peritoneal cavity from the spleen [23]. Hence it would be important to compare changes in different types of cell populations in the peritoneal cavity with the changes that may occur in the spleen as well as peripheral blood during the period of the immunization process. Since harvesting spleen cells and peripheral blood would require different protocols, these aspects would be considered in future studies.

Functional importance of peritoneal cells and protection against viral infections has been shown in a study performed in BALB/c mice using H1N1 virus infections [16]. In the peritoneal cavity, a 1.4 fold increase in the numbers of B-1 cells (1.4 fold) has been observed at 14 days post-infection compared to normal mice. Further, IgG + B-1 cells and IgG + B-2 cells were increased at 14 days post-infection. Virus induced virus-reactive IgG production in the peritoneal cavity in mice has been attributed to the cross-protection against a lethal intraperitoneal dose of H3N2 virus. The protective immunological responses induced against virus infection in the peritoneal cavity of mice suggest a potent immunoregulatory activity against influenza viruses which emphasize the importance of intraperitoneal route as a better selection of vaccination routes. Whether these findings are applicable to other immunization or infection against other infectious diseases remains to be further investigated. The role of CD8 + T cell memory in response to sequential priming or reinfection and increased T cell population after repeated H1N1 virus infection has also been highlighted as a mechanism for generating immunological memory in this system. This has some relevance to the findings from the present study where we observed a continuously increasing trend in the proportion of T cells in the group of rats immunized with serovar Pyrogenes. This emphasizes the needs for further studies with more specific markers for different T cell populations.

Another reason for the changes in B cell populations may be due to apoptosis and this has been reported during both viral and well as bacterial infections [16, 28]. Further, under normal conditions the elimination of low affinity cells favors outgrowth of the remaining high-affinity clones, and this is mandatory for the generation of proper antibody responses [29]. The significant increase in B2 cells in both groups of rats was expected which correlated with the humoral immune response, including both IgM and IgG antibody responses.

## Conclusions

The comparison of the two types of antigen preparation indicated that the LPS extracts of *L. interrogans* serovar Pyrogenes induced higher IgM responses while the IgG response was equivalent among the four antigen preparations tested. Among the lymphocytes accumulated within the peritoneal cavity, B2 cells significantly increased during the immunization period and positively correlated with IgM and IgG responses. Overall, the findings reflect that the two intraperitoneal immunization induced effective immune response and the peritoneal B2 cells may have played a considerable role in generating this humoral response against *Leptospira* antigens.

## Materials and methods

### Ethics statement

Ethics approval for the study was obtained from the Ethics Review Committee, Medical Research Institute of Sri Lanka (Ref No: 30/2016).

### Experimental animals

Female adult Wistar rats (4–6 weeks old) were purchased from Medical Research Institute (MRI), Colombo, Sri Lanka. The weights of the rats were measured, and they were acclimatized for a week as recommended [30]. Sample size was determined using resource equation method [31]. However, not having equal number of animals for assessment of immune response against sonicated antigen is a limitation of the study. They were grouped randomly and housed in clean polypropylene cages in the animal house of Institute of Biochemistry, Molecular Biology and Biotechnology (IBMBB), University of Colombo, Sri Lanka. The temperature was 28–31 °C, photoperiod approximately within a 12-hour light / dark cycle and relative humidity 50–55%. Ratswere fed with pellet food and clear drinking water [30]. At the end of the experiment, all rats were euthanized by intraperitoneal injection of mixture of Ketamine (250 mg/kg) /Xylazine (25 mg/kg) [32].

### Bacteria cultures

*Leptospira* cultures were maintained using Difco™ *Leptospira* Medium Base Ellinghausen-McCullough-Johnson-Harris (EMJH) medium which was prepared with 10% enrichment according to the instructions of the manufacturer. Cultures were maintained for 14 days in dark at RT to obtain a high density followed by sub-culture under aseptic conditions. One saprophytic *Leptospira* serovar; *Leptospira biflexa* serovar Patoc and one pathogenic serovar; *Leptospira interrogans* serovar Pyrogenes were used in this study [33, 34].

For quantification of bacterial cultures, McFarland standard series ranging from 0.5 to 4.0 was prepared by mixing 1.175% Barium Chloride with 1% sulfuric acid. Absorbance was taken at 600 nm spectrophotometrically (Synergy HTX Multimode Microplate Reader, Biotek, USA) and the standard curve was plotted against the McFarland standard. Absorbance of the cultures were measured in the same way and the bacterial density was determined using the equation derived from the McFarland standard curve [35].

### Preparation of sonicated antigen for immunization

Separate antigen preparations were made from both *Leptospira* serovars used. Bacterial cultures adjusted to the density of 0.9–1.2 × 10^9^/ml were centrifuged at 10,000 *g* for 30 min at 4 °C. The pellet was washed twice with 0.13 M phosphate buffered saline (PBS) (pH 7.2) and re-suspended in 25% of the original volume in PBS. The suspensions were sonicated for 3 times at 20 kHz for 3 min. The sonicated antigen preparations were stored at -20 °C in aliquots. Protein content was determined using Bradford method [36]. Antigen preparation from 1 × 10^7^ bacteria was used for each immunization. The antigen preparations made from the two serovars will be referred to as sonicated serovar Patoc and sonicated serovar Pyrogenes.

### Extraction and purification of *Leptospira* LPS

*Leptospira* LPS were extracted using a commercially available LPS extraction kit from iNtron Incorporation, South Korea according to manufacturer’s instructions [37]. LPS extracts were quantified using phenol–sulfuric acid method [38]. The LPS preparations made from the two serovars will be referred to as LPS-Patoc and LPS-Pyrogenes.

### Immunization of rats using sonicated antigen preparations

Wister rats were immunized with the two sonicated antigens, i.e. Sonicated serovar Patoc (n = 2) and sonicated serovar Pyrogenes (n = 2) and the two LPS extracts from the two serovars LPS-Patoc (n = 6) and LPS-Pyrogenes (n = 6). Three doses of antigen preparations were administered intraperitoneally at 2 week intervals. Each dose was suspended in 0.5 ml of 0.13 M PBS and emulsified in an equal volume of adjuvant mixture which consisted a ratio of 1:1 and 1:4 of Freund’s Complete Adjuvant (FCA) and Freund’s Incomplete Adjuvant (FIA) for the 1st and 2nd immunizations respectively. Third immunization was done using only FIA [39]. Adjuvant control group was administered with PBS mixed with FCA and IFA using the same adjuvant formulation. Pre-immune sera were separated from the blood collected on day 0 and immune sera were separated from weekly blood collected until day 35, through tail bleeding. Sera were stored at -20 °C until use.

### Assessment of Anti-leptospira IgM and IgG responses

Serum samples were screened for anti-leptospiral immune response using two in-house indirect ELISAs described by Niloofa et al. 2021 with slight modifications [40]. Briefly, sonicated antigen preparations (2 µg/ml) and LPS extracts (5 µg/ml) of each serovars were prepared separately to coat the ELISA plates using 0.05 M carbonate-bicarbonate buffer (pH 9.6). Plates were incubated overnight at 4 °C and washed 6 times with PBST, 0.5 M 1X PBS containing 5% milk was used for blocking. Plates were washed again for six times, respective serum dilutions were added to the plate and incubated for 2 h at 37 °C. Goat anti-rat IgM (µ chain specific)/ IgG (γ-chain specific) HRP conjugated secondary antibody dilutions (100 µl of 1:8000) were added to the plates and incubated for 1 h at 37 °C. After six washings, 100 µl of 3, 3, 5, 5- tetramethyl benzidine substrate dissolved in dimethyl sulfoxide and phosphate citrate buffer (pH 5.0) were added. Hydrogen peroxide was added as the catalyst. After 30 min of incubation, the reaction was terminated by adding 1 N HCl. The optical density was obtained at 450 nm using multimode microplate reader (Synergy HTX Multimode Microplate Reader, Biotek, USA).

### Assessment of peritoneal lymphocyte subpopulations by flow cytometric analysis

Peritoneal cells were harvested on day 0, 7, 14, 21 and 28 for analysis of cells subpopulations by flow cytometry. Rats were kept in ventral positions and 30 ml of sterile 0.13 M PBS was injected into the peritoneal cavity using the 23 G needle and 18 G cannula was used to drain the peritoneal fluid. Extracellular staining protocol for flow cytometry was optimized to analyse rat peritoneal cells. NaN_3_ and ice-cold reagents were used to prevent the modulation and internalization of surface antigens during the staining procedure. The cell suspension was adjusted to a concentration of 1.0 × 10^6^ cells/mL with cold (4 ^o^C) PBS/ 3% BSA. PE conjugated anti-rat CD3, APC conjugated anti-rat CD45RA and PerCP/Cy5.5 conjugated anti-rat CD11b diluted according to manufacturer’s instructions were used to stain cells. Antibodies were added to 100 µl of cells suspensions, mixed well and incubated at 4 ^o^C for 30 min in dark. Cells were washed three times with 1 ml of cold PBS/ BSA followed by centrifugation at 400 *g* for 5 min at 4 ^o^C. The cells were re-suspended and placed in the dark, at 4 ^o^C prior to flow cytometric analysis. The fixed cells were analyzed using flow cytometer (BD FACS Calibur flow cytometer, BD Bioscience, USA) within 24 h. Unstained controls were used to monitor auto-fluorescence. Fluorescence Minus One (FMO) control was used to compensate the overlapping emission spectrums, which assist to identify gating boundaries in multi-color flow cytometry. The data acquired were analyzed using a strategy of gating the lymphocyte population from the density plot of forward and side scatter profile and further gated to T, B, B1 and B2 cell populations using antibodies against CD3, CD45RA and CD11b [41]. Different peritoneal cell subpopulations were assessed as the percentages of mononuclear phagocytes (*SSC-H*^mid^, *FSC-H*^high^, *CD11b*^high^*)*, granulocytes *(SSC-H*^high^, *CD11b*^mid^*)*, lymphocytes *(SSC-H*^low^, *FSC-H*^high^ ), T cells (CD3^high^), B cells (CD45RA^+^), B1 cells (CD45RA^+^, *CD11b*^Mid^) and B2 cells (CD45RA^+^, *CD11b*^low^). Gating strategy is depicted in Supplementary Fig. 1.

### Data analysis

Statistical analysis was performed using IBM Statistical package for Social Sciences (SPSS) version 26. None of the animals were excluded from data analysis. Parametric and Non- parametric analysis were followed based on the distribution of data by using Shapiro-Wilk test and skewness and Kurtosis values. Absorbance was presented as mean ± SD. Correlations were analyzed using Pierson and Spearman correlation tests. Differences were considered as statistically significant at p ≤ 0.05 at 95% confidence interval. * p < 0.05 was considered significant, ** p < 0.01 was considered markedly significant. Multiple comparisons between groups were performed using Tukey’s post-hoc test.

### Electronic supplementary material

Below is the link to the electronic supplementary material.

Supplementary Material 1

## Data Availability

The data used to support the findings of this study are available from the corresponding author upon request.

## Abbreviations

LPS: Lipopolysaccharide
PC: Peritoneal cavity
HSCs: Heamtopoietic stem cells
GPR183: G protein-coupled receptor 183
EMJH: Ellinghausen-McCullough-Johnson-Harris
PBS: Phosphate buffered saline
FCA: Freund’s Complete Adjuvant
FIA: Freund’s Incomplete Adjuvant
