# Supplementary material for: Humoral immune response and changes in peritoneal cell populations in rats immunized against two Leptospira serovars; serovar patoc and serovar pyrogenes
Source: BMC Immunol. 2023 Oct 17;24:39. doi: 10.1186/s12865-023-00574-z (PMC10583450; doi:10.1186/s12865-023-00574-z)
Supplement: Supplementary file 1 — Supplementary Material 1 [file 12865_2023_574_MOESM1_ESM.docx]

**Supplementary Table S1: Estimated cell numbers in animals immunized with LPS-Patoc and LPS-Pyrogenes from day 0 to day 28**

| **Cell type** | **LPS Immunization group** | **Cell number on** | | | | |
| --- | --- | --- | --- | --- | --- | --- |
|  |  | **Day 0** | **Day 7** | **Day 14** | **Day 21** | **Day 28** |
| Total peritoneal cells assessed | Patoc | 94,500 | 91,300 | 83,000 | 96,600 | 94,500 |
|  | Pyrogenes | 94500 | 91,100 | 97,200 | 97,800 | 92,100 |
| Lymphocytes | Patoc | 46,251 | 48,851 | 68,519 | 85,579 | 55,657 |
|  | Pyrogenes | 46,251 | 61,648 | 43,755 | 90,349 | 43,764 |
| *Percentage of Lymphocytes (%)* | *Patoc* | *48.9* | *53.5* | *82.6* | *88.6* | *58.9* |
|  | *Pyrogenes* | *48.9* | *67.7* | *45.0* | *92.4* | *47.5* |
| T cells | Patoc | 33,226 | 28,978 | 46,523 | 65,864 | 40,107 |
|  | Pyrogenes | 33,226 | 50,062 | 35,490 | 75,878 | 38,135 |
| *T cells as a percentage of lymphocytes (%)* | *Patoc* | *71.8* | *59.3* | *67.9* | *77.0* | *72.1* |
|  | *Pyrogenes* | *71.8* | *81.2* | *81.1* | *84.0* | *87.1* |
| B2 B cells | Patoc | 727 | 2,198 | 2,519 | 3,660 | 4,085 |
|  | Pyrogenes | 727 | 1,197 | 1,070 | 3,095 | 1,795 |
| *B2 cells as a percentage of lymphocytes (%)* | *Patoc* | *1.6* | *4.5* | *3.7* | *4.3* | *7.3* |
|  | *Pyrogenes* | *1.6* | *1.9* | *2.4* | *3.4* | *4.1* |

**Supplementary Figure 1:**- Gating strategies for flow cytometry

.

Peritoneal cell population

Lymphocytes - *(SSC-H*^low^, *FSC-H*^high^ )

MNPs – (*SSC-H*^mid^*, FSC-H*^high^*, CD11b*^high^)

Granulocytes *(SSC-H*^high^*, CD11b*^mid^*)*

B cells- CD45RA^+^

T cells – CD3^high^

B2 cells (CD45RA^+^, *CD11b*^low^)

B1 cells (CD45RA^+^, *CD11b*^Mid^)
